# Assessment of Ecological and Toxicological State of Soils and Waters in the Neighborhood of Mining Industry Enterprises in the Armenian Highlands

**DOI:** 10.3390/life13020394

**Published:** 2023-01-31

**Authors:** Meruzhan Haykaram Galstyan, Maxim Viktorovich Larionov, Hovik Yakhsibek Sayadyan, Karine Shahen Sargsyan

**Affiliations:** 1 Faculty of Agronomy, Armenian National Agrarian University, 74 Teryan Street, Yerevan 0009, Armenia; 2 Faculty of Ecology and Environmental Protection, Russian State Social University (RSSU), 4 Wilhelm Peak Street, Building 1, 129226 Moscow, Russia; 3 World-Class Scientific Center “Agrotechnologies for the Future” (CAAT), Russian State Agrarian University—Moscow Timiryazev Agricultural Academy, 49 Timiryazevskaya Street, 127550 Moscow, Russia; 4 Institute of Industry Management, State University of Management (SUM), 99 Ryazanskii Prospect Street, 109542 Moscow, Russia; 5 Federal State Budgetary Educational Institution of Higher Education, “State University of Land Use Planning” (SULUP), 15 Kazakov Street, 105064 Moscow, Russia; 6 Faculty of Geography and Geology, Yerevan State University, 1 Alec Manukyan Street, Yerevan 0025, Armenia

**Keywords:** mining industry, wastewater, contamination, heavy metals, toxicants and supertoxicants, chemical composition of waters, agrochemical status of soils, condition of soils and waters, sanitary and hygienic safety, environmental safety, artificial ecosystems, organic farming, productivity of potatoes, beans, eggplants, ameliorants, nutrients, state and productivity of phytocenoses, optimum development of plants, environmental and hygienic safety, food security, Sotk river, Masrik river, Karchaghbyur river, Lake Sevan, Armenian Highlands

## Abstract

The article presents the results of the seven-year study on the state of arable lands and wastewaters in the districts of mining enterprises in Armenia. An assessment of the ecological and toxicological state of wastewaters and contaminated areas was carried out. Methods for their purification are proposed aimed at their further use and obtainment of environmentally safe agricultural products. An area of about 0.5 ha, next to the rural community of Syunik in southern Armenia, has been polluted for many years by mining sludges from the nearest mine watertight cofferdam of the Zangezur copper-molybdenum combine. Activities have been carried out to clean up the soil in this area. After plowing, soil improvers (zeolite, bentonite, and manure) were introduced into the soil. On-site treatments, soil tillage, and the introduction of soil improvers into the soil was implemented in late autumn. Soil and plant samples were taken to determine the content of heavy metals (Cu, Zn, Pb, Co, Mo, Ni). In the following spring, the area was planted with potatoes, eggplant, and peas. A rather high yield was obtained. Analysis of plant samples showed that the content of heavy metals does not exceed the permissible level of the international food safety standard. At the same time, soil studies were carried out in the adjacent areas of the Sotk mine, located to the southeast of Lake Sevan. It was revealed that due to the increase in the volume of the mining industry and the amount of rock dumps, the organoleptic and chemical indicators of the waters of the Sotk and Masrik rivers deteriorated. Compared to the previous decade, the amounts of suspended particles per 1 L of water have increased by 210…317.0%, in the waters of Sotk—by 32.1 mg/L, and in the waters of Masrik—by 13.2 mg/L. The same tendency is observed regarding the amounts of nitrates, sulfates, and hydrogen index, which is mainly due to the chemical composition of rocks. They contain a large amount of Ca, K, Na, Mg, etc. This trend is especially noticeable along the rivers, where intensive agriculture, primarily livestock farming is carried out. The material of the work solves a complex of environmental and economic problems. It is aimed at ensuring environmental safety, improving the ecological and resource properties of soils, increasing the productivity of cultural phytocenoses and the sanitary and hygienic quality of food products.

## 1. Introduction

The mining industry causes great harm to the environment. Practically all living environments are significantly polluted. It is indicated that in a number of regions of the world there is an increase in the role of industrialization of the extractive industry and an increase in technogenic pressure on the environment [1,2]. Of course, many sectors of the national economy harm the environment [3,4,5,6]. However, the negative environmental consequences of mining are many times higher [7,8,9,10].

The mining industry is one of the more developed industries in the Republic of Armenia (RA). At the same time, it produces large volumes of toxic waste and pollutes the environment, damaging ecological systems and the health of the population living near mining sites. For example, the operations of these mines affect the quality, quantity, and safety of agricultural products grown on lands neighboring mining sites and the mining industry’s waste dumps. The environmental health protection apparatus of Armenia allows mining enterprises to discharge highly toxic wastewater/sludge into surface waters without treatment, thus polluting the streams and rivers with heavy metals and acid mine drainage. At the same time, the water of these same streams and rivers is used for irrigating fields and orchards. In addition, mine tailings dams (“reservoirs”) are poorly designed and constructed, such that there are frequent spills from the dams; the dams are not isolated from the environment (open to the air, leaking from the floor and sidewalls, etc.).

Currently, Armenia has over fifty thousand hectares of metal-contaminated land, thirty thousand of which are in the north-east of the country, covering forest soils and river valley soils, where the concentration of heavy metals exceeds the natural background by 3.5–40.6 times [11,12,13,14].

Heavy metals (Cu, Zn, Pb, Co, As, Mn, Ni, etc.) contaminating soil, groundwater, and surface waters, eventually make their way into plants, where their toxicity affects the physiologic functions of the plant, such as growth, productivity, and product quality. In humans, these metals cause various forms of cancer, sterility, respiratory and other diseases [15,16,17,18,19,20,21,22].

**Figure 1:**
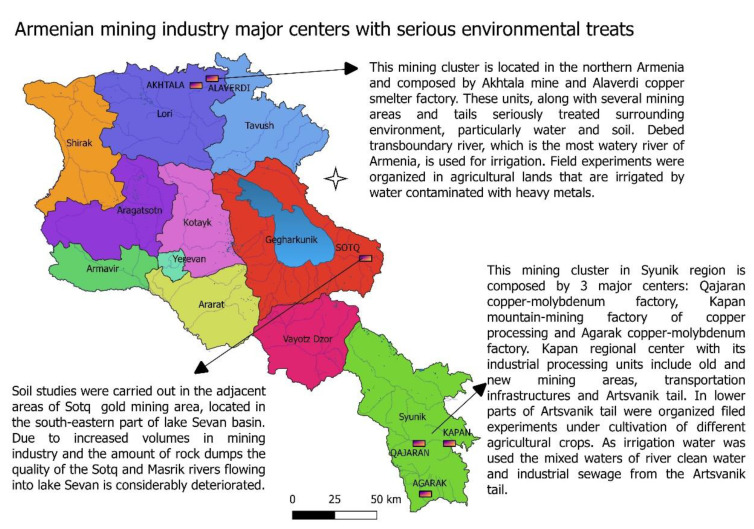
Armenian mining industry locations.

The lands, in proximity to the mining operations at Qajaran, Kapan and Sotk, are rendered useless for many reasons—mine tailings spills, mine explosions producing clouds of toxic dust which settles on land, use of irrigation water contaminated with heavy metals, toxic dust from the surface of tailings dumps dispersed through wind action, direct dumping on the land of various mine wastes, etc. (Figure 1).

We believe that the development of measures aimed at salvaging these lands to produce environmentally clean agricultural crops, by ameliorating the soil with amendments, which improve soil structure, immobilize the heavy metals making them unavailable for the plants, and, at the same time, fertilize the soil to raise the overall productivity of the land, is of paramount significance, relevant and stem from the national security of the population.

## 2. Materials and Methods

To evaluate the qualitative and quantitative impacts of the RA large mining sites, particularly those of Kapan, Qajaran and Sotk provinces on the neighboring agroecosystems as well as to assess the ecological state of the waters of Sotk and Masrik rivers flowing along the vicinity of the Sotk gold mine and their technogenic burden, their contamination level has been determined, soil cutting has been conducted, sampling of tailings and the soil thereunder has been done, the content of heavy metals and soil agrochemical indicators (pH, humus content, carbonates, mobile nutrients) have been determined.

Several spots were selected randomly on the tailings layer for sampling using a soil auger and sampled at a depth of 0–20 cm. After the tailings layer was removed, the plot was fenced off, 5 spots were selected within the cleared square plot and sampled at a depth of 0–25 cm with soil auger. All samples were taken to the laboratory and dried (100–105 °C), which allowed the determination of water content by measuring wet and dry sample weights. After drying, samples were ground to pass through a 2 mm sieve. For each—tailings layer and the soil—one composite sample was created by mixing of the samples taken from several spots. Total heavy metal content was assessed in each composite sample using strong acid (HNO_3_) digestion–extraction, and afterwards using atomic absorption spectrophotometer. The mobile or leachable heavy metal fraction was extracted using a buffer solution of acetic acid–ammonium hydroxide adjusted for pH 4.8. The samples were left at room temperature in the buffer solution for 24 h—soaking and shaken 5–7 times during the soaking period. After 24 h, the solution was shaken again and filtered. The mobile or leachable forms of heavy metals in the filtrate were quantified using atomic absorption spectrophotometer.

pH of the soil and tailings samples were estimated by dipping the pH electrode meter in the saturation paste. The sample content of humic substances was determined using Tiurin’s method for the determination of organic carbon (using titration with phenyl anthranilic acid). Total carbonate content was quantified by acid dissolution and by measuring the subsequent release of titrimetric CO_2_.

Water-soluble Nitrogen in the samples was extracted and determined using the method of I.V. Tiurin and M.M. Kanonova (USSR scientists); mobile phosphorus was determined using Machigin’s method, while the exchangeable potassium was quantified using Maslova’s method (both USSR scientists).

Concentrations of heavy metals in crop samples were determined as follows: plant samples were dried, ground, and treated with strong acid digestion-extraction, afterwards the metals’ content was determined using atomic absorption spectrophotometry (AAS–1N and AAS–30) CENS (Center for Ecological-Noosphere Studies of the National Academy of Sciences. For potatoes, starch content was also assessed through the weighing method, and the nitrate content was determined through “COEX” nitrate meter [23].

The studies have been conducted through water sampling from the appropriate observation points of the Sotk and Masrik rivers situated near the Sotk mine and through determining their organoleptic and chemical indices. It has been planned to determine the dynamics of the investigated elements by comparing the obtained data with the similar indices of the water samples taken from the same observation point by the Environmental Monitoring and Information Center (SNCO) under the Ministry of Environment within the previous 10 years and to accordingly develop the needed recommendations.

Considering the circumstance that reoxygenation, as well as physicochemical, and biochemical processes and intensive microbial activities, sorbtion, desorbtion, and other activities, are taking place in water, besides, the water organoleptic properties can change, the laboratory studies on taste, flavor, color, permeability have been conducted with the standard methods [24,25]. Hydrogenic index, specific electrical conductivity, and salinity have been determined through the electro-chemical method: the total dissolved substances have been calculated by multiplying the value of specific electrical conductivity by 0.65.

Biochemical oxygen demand (BOD) represents the amount of oxygen (mg) needed for oxidizing organic matters in 1-L water at 20 °C under aerobic conditions over some period: in our case it has been determined for 5 days [24,25].

Biochemical oxygen demand (BOD) (biochromatic oxidation) has been determined in the acidic medium of potassium bichromate under the guidance of catalyzers’, while titration has been implemented with 0.025 N solution of the Mohr’s salt. It has been determined through the following formula: Cx = (n_1_ − n_2_) × 8 × V_2_/V_1_ × 1000, where V_1_ is the volume of the investigated water, V_2_ is the volume of the potassium bichromate, n_1_ is the volume of the Mohr’s salt when titrating the zero sample and n_2_ is the volume of the Mohr’s salt when titrating the sample. In drinking water, the rate of BOD makes 15 mg O_2_/L, while in sanitary and irrigation waters it makes 30 mg O_2_/L [25]. Ammonium, silicium, nitrate, and phosphorus ions have been determined with KFK-2 spectrophotometry (Shimadzu 1650) at the wavelength of 360–600, 410, 536, and 708 nm respectively. Sulphate, chloride, nitrate ions have been determined through the ion chromatography method (DIONEX-1000), while the hydro-carbonate- through the back-titration method [25]. 

The permeability, flavor, and color are technical criteria, which have been determined through visual and sensory methods. The analysis of the chemical elements (Li, Be, B, Mg, Na, Al, P, K, Ca, Ti, V, Cr, Fe, Mn, Co, Ni, Cu, Zn, As, Se) in the water samples have been conducted through the inductively coupled plasma mass spectrometry (ICP-MS, ICP-MSELAN 9000) in line with the standard of ISO 17294, which is based on the use of argon inductively coupled plasma as a source for ions and the use of mass spectrometry for ion separation and their further determination [24,25,26,27,28,29,30,31,32,33,34,35,36]. The field experiments have been conducted for the three crops (potato, bean, and eggplant).

## 3. Results and Discussion

The field experimental activities were conducted in 2013–2014 on the brown forest soils of the Syunik region, near the tailing dam base of the mining combine situated in the community of Syunik, which is 729.4 m high above sea level. The territory was covered on average with a 20 cm tailings sludge layer. In the autumn of the previous year (2013) the area where further field experiments were set up, was completely cleaned up from the sludge layers of the tailings and all the necessary preparatory activities were implemented per the patterns of the trials which are introduced in appropriate tables; the natural mineral ameliorants—zeolite and bentonite (transported from Noyemberyan and Ijevan provinces) and half-rotted manure with corresponding doses were introduced in autumn per the experimental scheme in the period of main soil treatment activities, while the crop sowing and cultivation activities were implemented in 2014 in accordance with the agricultural rules adopted and applied for that zone.

Harvesting was conducted based on the crop ripening degree upon the general calculation method. The results of laboratory investigations of the tailings sludge and its underneath soil have shown, that the tailings sludge has a weak alkaline reaction (pH 7.45), the content of carbonates makes 12.1% of the whole mass, where humus and plants available nutrients (NPK) are missing (Table 1) and the data of the same table indicate that in the soil under the tailings (20–45 cm) the humus content makes 1.27%, while the content of plants available nutrients—nitrogen, phosphorus, and potassium, though insignificant, respectively make 0.85, 0.67 and 13.02 mg in 100 g soil.

The contamination level of the tailings sludge and its underneath soil is also different (Table 2). As the table data show the content of both the total and mobile forms of heavy metals is very high and in case of such conditions no agricultural crop can ever grow and develop properly.

The table data show that the tailings contain high levels of heavy metals (Pb, Ni, Cu, Zn) and their mobile forms, while the soil below contains considerably fewer metals compared to the tailings layer; the content of total heavy metals in the underlying soil layer is 7.9–21.0 times less, while the mobile forms are 3.1–48.0 times less than those in the tailing’s layers.

The measures taken by our research group were related to the mentioned circumstance: first, the tailings sludge was removed to create favorable conditions for the crop’s growth and development, and then the afforested field experimental studies were conducted.

In the autumn of 2013, after introducing manure and ameliorants in the experimental field 24 soil samples were taken from the area allocated for he mentioned three crops before the sowing activities in the upcoming spring; they were then treated, and on average eight samples were produced, thereafter the content of mobile forms of heavy metals was determined in order to find out how the manure and ameliorants affected the amounts of mobile forms of heavy metals within the mentioned 6 months starting from autumn to spring. Eventually, the recorded changes were compared with the indices of the control (without fertilization) variant (Table 3).

Table 3 data show, that manure and ameliorants have not caused considerable changes in the content of heavy metals within the autumn-spring period. This is probably due to the circumstance, that within the period of introducing manure and ameliorants and the crops sowing time which coincides with cold weather and overdamping season, no microbiological processes took place and as a result, no interactions of the manure and ameliorants with heavy metals was observed [35]. 

The results of field and laboratory research have indicated that both manure and natural mineral ameliorants applied on the background of manure have somehow affected the growth and development as well as the yield amount and qualitative indicators of potato, bean, and eggplant crops. In the humus-poor soils of the experimental plot weakly provided with the available nutrients (NPK), the crops of the experimental variants, where manure and various doses of zeolite and bentonite on the background of manure were applied, have grown more intensively as compared to those in the variant without fertilization (control); during the vegetation period, they were always characterized with dark green color and consequently provided higher yield than the crops of the control variant (Table 4).

The data of Table 4 show, that in the variant of manure 40 t/ha (background) the potato yield capacity was 199.0 c/ha, green bean—94.0 c/ha, eggplant—175 c/ha (according to the three-time harvesting result per the ripening degree), in the control variant the potato, bean, and eggplant yields made 100.6, 67.0 and 87.0 c/ha respectively, then in case of applying 3 t/ha, 6 t/ha and 9 t/ha zeolite and bentonite on the same background, the yield of potato tubers against that of the background variant increased by 14–28 c/ha (in case of zeolite) and by 50–79 c/ha (in case of bentonite), the yield of bean accordingly by 3–10 c/ha and 21–38 c/ha and eggplant yield—by 9.0–28.0 and 38.0–54.0 c/ha respectively.

At the same time, the analysis of yield capacity results discloses that though the application of equal doses of zeolite and bentonite on the same background has increased the yield capacity in all experimental variants, bentonite has provided still higher yield than zeolite: for potato fields, it exceeded by 37.8–50.7%, in case of bean—by 17.6–31.5% and in eggplant sowings—by 16.0–29%. This difference is accounted for by the fact that first of all zeolite is endowed with an alkaline reaction-forming ability and the soils of this region are characterized by a weak alkaline medium (pH—7.35–7.45), thus, under such conditions, the alkalinity grows up even more suppressing the growth of both aboveground and underground parts of the plants; besides, zeolite contains very little amount of nutrients needed for the plants (total phosphorus—2.2–2.7%, total potassium—0.12–0.23%). Contrarily, bentonite has a neutral environmental reaction (6.8–7.1), where the content of phosphorus, potassium, and magnesium is 0.18%, 1.2%, and 3.62% respectively; besides, it is most likely that in this circumstance it also serves as a growth stimulant [14,16,37,38,39,40,41,42,43,44,45].

The data of Table 4 clearly state that the yield capacity of the agricultural crop increases together with the increase of zeolite and bentonite doses on the background of manure, yet the best variant proves to be the dose of 6 t/ha, since the further increase in the doses of ameliorants though increased the yield capacity of potato, bean and eggplant, nevertheless the increased amount was not significant and fluctuated within the range of experimental error (S_x,_ %) and the least significance difference (LSD_0,95,_ c). The relevant methodology [46] and relevant statistical analyses package was used [47].

**Figure 2:**
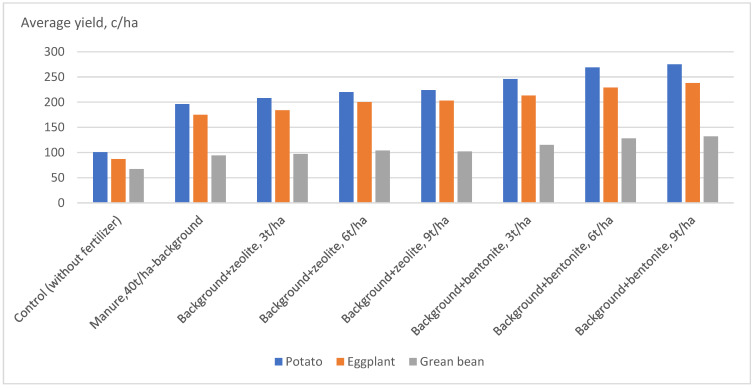
The effect of applying growing doses of natural mineral ameliorants on the background of manure on the crops qualitative indicators.

Meanwhile, the laboratory investigations have shown that though compared to the control variant the nitrate content per kg of dry matter has increased in the yields of potato tubers, green bean, and eggplant in both background (manure 40 t/ha) and background + zeolite, bentonite variants by 80–100 mg for potato, by 88–151 mg for eggplant and by 60–95 mg for green bean, yet the amounts of nitrates in all crops were within the maximum permissible concentration limits (Table 5, Figure 2). Below the same table, the MPC values of nitrates for the studied crops are introduced.

In the case of 40 t/ha manure application during the experiment the content of nitrates increases by a little amount, since, as has been already mentioned, the soils of the experimental plot are endowed with extremely low fertility, the humus content in the topsoil does not exceed 1.5% and they are poor in easily hydrolysable nitrogen. Under such conditions, the nitrogen of the manure together with other nutrients was used by the crops to produce above- average yield for the given agricultural zone. The same regularity is also observed in the case of starch content in the potato tubers, the content of which has hardly changed in the variant where manure has been introduced as compared to that of the control variant (in the control variant the starch content makes 17.5% and in all other variants it is 15.9–17.0%), moreover, the surplus of starch yield against the control variant is related to the high yield of potato tubers [11,14,16,37].

The results of laboratory investigations have shown that the application of both manure and zeolite and bentonite doses against the manure background has exerted a certain effect on the accretion of heavy metals (Pb, Ni, Cu, Zn, Co) in the yield of potato tubers and green bean (Table 6). If we compare the data of the same Table 6 with those on the content of mobile forms of heavy metals before the crop planting per variants introduced in Table 3 it becomes clear that the content of all heavy metals in all variants, even the content of lead (Pb) has not exceeded the normative amounts and they are within the maximum permissible concentration limits.

Such low amounts of mobile forms of heavy metals are first of all due to the measures taken related to the removal of the tailings sludge layer in autumn (averagely 0–20 cm) and then to the introduction of manure and ameliorants into the top soil, as a result of which some part of mobile forms of soil heavy metals together with the organic substances of the manure form hardly soluble compounds (chelates) for the root system of the plants, while the other part joins the micellar (crystal) network of zeolite and bentonite due to their high adsorption capacity and as a result, the amount of heavy metals are left in the soil, which is needed as microelements to organize regular plant nutrition during the vegetation period.

Nevertheless, upon the impact of manure, zeolite, and bentonite the migration of heavy metals to the plant organism during the vegetation period has been reduced and, as a result, the contents of all heavy metals, including Lead (Pb) in the yield of potato tubers and green bean comply with the requirements of food ecotoxicity regulations and are in line with all standards for food safety.

It is also noteworthy, that the absolute value of the content of zinc and nickel in the yield of green beans against the same elements found in the potato tubers is accounted for the selectivity and accumulation properties which are characteristic, particularly to leguminous crops.

At the same time upon the results of investigations, it has been disclosed that the organoleptic indices, (permeability, suspension particles, color, and flavor) both in the Sotk and Masrik rivers, are different depending on the sampling times. The water quality deteriorates starting from May up to November, which is related to the rain and meltwaters, as well as to the intensive atmospheric activities. It is apparent that a smaller number of suspended particles in the waters of the Sotk and Masrik rivers are found particularly in the water samples taken from the territories closer to the mining site. Thus, in November (2019), the suspended particles in the water samples taken from the territory situated 1 km above the Sotk community and opposite the community have made 3.5 mg/L and 11.5 mg/L respectively (Table 1), while in the waters of the Masrik river 1.5 km above V. Shorzha community and opposite the mentioned community it has made 5.5 and 19.9 mg/L respectively; at the mouth of the Sotk and Masrik rivers and after interflowing of the waters their suspended particles amounted to 47.5 and 28.7 mg/L respectively (Table 7 and Table 8).

If we compare the mentioned indices with the similar average date of the last previous 10 years, it becomes obvious that along with the increase of the mining industry dimensions, as well as with the increase of the rock dumps amount, the quantity of the suspended particles have also grown up and, as to the mentioned studies, the amounts of the suspension particles per liter of water have increased by 210.2–317.0% as compared to the water samples taken within the same period in 2010, or to be more precise, in waters of the Sotk river they have increased by 32.10 mg/L and in waters of the Masrik river—by 13.2 mg/L.

The suspension particles have a great impact on the water permeability, its color, flavor, as well as on its biological and bio-chemical indicators, which is more clearly described in the tables. Electrical conductivity is one of the vital ecological indicators of surface waters. As we can see from the data in Table 7 and Table 8 provided by the studies of the Environmental Monitoring and Information Center, the values of electrical conductivity in the Sotk and Masrik rivers grow up for the period of December–March compared to those observed for the period of Spring-Autumn. The mentioned data testify that in the winter hydrological period the river flow results from the feeding of ground and underground waters and their hydro-chemical quality is formed under the hydro-chemical influence of just the mentioned waters. Considering that, unlike the river waters, the values of electro-conductivity in the waters of cellar pits practically stay unchanged in that period and have 2–2.5 times higher values than those recorded in the river waters, it can be concluded that in the period of December-March the hydro-chemical composition in the waters of the Sotk river is affected also by the drainage waters of the mining site and cellar pits.

The results of the laboratory studies show that in the waters of the left-bank tributary of the Sotk and Masrik rivers, the feeding of which hardly has any relation with the Sotk mine, the average value of electro = conductivity is lower by 2–4 times the values generally recorded in the waters of the mentioned rivers. For comparison, the values of the electro-conductivity in the waters of the Karchaghbyur river mouth have been also studied, which do not have any seasonal variabilities and are 2 times lower than the average electroconductivity value observed in the waters of the Masrik river mouth.

The investigated waters are equivalent to the alkaline waters regarding the values of their hydrogenic indicators (pH). Thus, the pH in the Sotk river mouth makes 8.78, in the water sample taken from 1.0 km above the Sotk community it makes 8.43, while in the Masrik river mouth it is 8.38 and, in the samples, taken from the same river, 1.5 km above V. Shorzha community, it makes 8.21. This circumstance is related to the chemical composition of the environmental mountain ores, where the alkaline chemical elements (Ca, K, Na, Mg, etc.) are prevailing, the content of which has always been high both in the period of 2010–2015 and in the period of our investigations.

The Sotk river waters contain on average 30.2 mg Ca, 1.3 mg K, 5.5 mg Na and 31.6 mg Mg per liter of water, while in the waters of the Masrik river the mentioned indices have made 22.5; 2.0; 6.3 and 5.4 mg/L respectively. The content of Ca has made 26.00 mg/L, K—2.60, Na–6.45 and Mg—5.20 mg/L (Table 9 and Table 10), and the mineralization degree in the waters (total amount of the inorganic compounds) does not exceed 1 g/L and, per the average data of the previous 10 years, it makes 129.8 mg/L (in the waters of the Masrik river), 263.4 mg/L (in the waters of the Sotk river), for the period of our investigations (November, 2019) the mentioned index in the waters of the Masrik river has made averagely 146.9–182.0 mg/L and in the waters of the Sotk river it makes 230.8–253.5 mg/L. Besides, 95–96% are hydrocarbons; 4–5% are sulphates, while Se and Al make very little amount.

As to the content of nitrate, nitrite and ammonium ions it is clear from the table data that upon the results of the investigations conducted in the previous years and upon the laboratory investigations on the water samples conducted in November, 2019, they hardly exceed the standards set through the decision 75N on the surface waters assessment, taken on January 27, 2011, by the RA Government, which make NO_3_−—45, NO_2_−—3.3, NH+—2 mg/L and NO_3_−—0.02–1.5, NO_2_−—0.001–0.04, NH_4_+—0.03–0.7 mg/L respectively.

The data of EMIC (Environmental Monitoring and Information Center) testify that for the Winter-Spring period, in the waters of all observation points the concentration of nitrates grows up parallel to the intensification of water flow as well. In the waters of cellar pits, unlike in those of the rivers, the concentration of the nitrate almost stays the same and it is 2–4 times lower, and hence, the effect of the drainage waters in the cellar pits is insignificant, while there are more considerable factors forming the content of nitrates in the river waters, which are mostly related to the anthropogenic factors and to the fluctuation of the temperature. The increase of the nitrate content is mainly conditioned by the intensive agricultural activities implemented in the given areas, particularly in the riverine land areas, as well as, most likely, by the development of the livestock sector. As a result of the single mineral fertilization with nitrogen, some part of the nitrogen is leached and moved into the river mingling with the river waters. At the same time, some part of the cattle manure and liquid manure is imbibed into the soil and the other part is leached with the atmospheric precipitations and becomes mixed with the river waters increasing the nitrate content.

A similar pattern is observed in the case of the concentration dynamics in the sulphate ion, which also grows up along with the water flow intensification. Unlike the river waters, in the waters of the cellar pits, the sulphate concentration practically stays the same and is 2–3 times lower. So, the recorded sulphate content in the waters of the Sotk and Masrik rivers is formed under the influence of the Sotk mine exploitation, under the impact of pressure factors existing near the zone of the mining site along the south-eastern part of the Areguni mountain chain, as well as under the influence of the waters in the zone of the seepage flow (internal stream) of the Sotk and Masrik rivers, particularly under the significant impact of right-bank ground and artesian (underground) waters of the catchment basin.

While in the lower parts of the rivers the increase of the sulphate ion content is mainly related to the application of various agrochemicals during agricultural farming, as well as to the vaccines administered throughout cattle breeding, the residuals of which are poured into the rivers flowing through the mentioned territories increasing the amount of the mentioned ions.

The research results have indicated that the content of the heavy metals and other chemical elements in the Sotk and Masrik rivers is significantly low or are in line with the MPCL as to the data gained upon the investigations conducted in November 2019 and during the previous 10 years.

Besides, we will achieve a more comprehensive view of the above-discussed issue, if we put forward the circumstance that within the study period the total intensity of the geochemical flow of the heavy metals in the waters of the mentioned rivers significantly fluctuates and varies depending on the exploitation of the Sotk mining site and on the changes of the mining ore volumes. The results of the analysis of soil samples taken from the arable lands of agrocenosis in the studied river basin show that the lands irrigated with the waters of the Sotk-Masrik rivers have a high content of heavy metals in the soil samples, while the ploughlands irrigated with the waters of Karchaghbyur river almost no heavy metals have been detected (Table 11).

As the table data show the soils of the land areas irrigated with the waters of the Sotk and Masrik rivers (20–25 cm in arable lands) contain higher amounts of copper, zinc, lead, arsenic, mercury, and manganese, while the content of other metals is insignificant and is lower than the background indices of phytotoxic concentrations of those metals in the soil. Whereas in the land areas irrigated with the waters of Karchaghbyur river no content of the mentioned and other metals has been detected. This circumstance comes to prove once again that the rivers of Sotk and Masrik obtain direct feeding from the accessions of the Sotk mine and due to the leaching of dust and rock deposits formed during the mine exploitation (via precipitations), the heavy metals in the ores penetrate the river waters, which accrete in the soil plowing layer throughout the years. While in the soils of the land areas irrigated with the waters of the Karchaghbyur river heavy metals are missing, which is conditioned by the fact that the feeding of this river is not related to the mining site. As shown in the table, there are small amounts of As, Cu, and Zn in the waters of the Karchaghbyur river and in the soil samples taken from the lands irrigated with these waters, which are resulted from the residual amounts of pesticides used in agriculture, while the existence of Pb is due to the emissions of the vehicles operated in the mentioned region.

## 4. Conclusions

Studies of the content of heavy metals and other chemical elements in the waters of the Sotk and Masrik rivers and the soils of agroecosystems revealed that, as in the previous 10 years, the total geochemical intensity of their flow fluctuates within unacceptable Maximum permissible concentration (MPC) limits. This is due to the operation of the mine and changes in the volume of the ore mine. To improve the ecological and toxicological condition of the waters in these rivers and the soils irrigated therewith and to reduce the mobility of heavy metals, it is necessary to constantly increase the amount of CaO gypsum in the acid mine drainage so that the reaction of the aquatic environment is maintained within the range of at least 9 (pH = 9). At the same time, some heavy metals (wolfram, molybdenum, vanadium, etc.) are more active in an alkaline environment, so filters must be installed to purify water and soil.

To obtain ecologically safe food from the land areas covered with the sludge layer of mine tailings it is necessary to first clean and remove the sludge layers from those territories and only then implement cultivation activities of agricultural crops.

Since the soils buried under the tailings layer have a very low level of fertility, it is necessary to fertilize the growing crops with 40 t/ha manure, while in order to reduce the migration of heavy metals from the soil into the plant organism and to produce food meeting the requirements of eco-toxicological standards and pose no hazard to human health, zeolite or bentonite of 6 t/ha application dose with manure should be introduced in the technogenic contaminated soils.

Along with the increase of the mining dimensions in the Sotk gold mine and with the increase of the amounts of rock dumps, the organoleptic and chemical indicators in the waters of the Sotk and Masrik rivers also grow up. The quantity of the suspension particles has increased by about 210–317.0% as compared to the same indices obtained for the last 10 years (2010); in the waters of the Sotk river, the increase has amounted to 32.1 mg/L, while in those of the Masrik river it makes 13.2 mg/L.

Increasing tendencies have been also observed in the mentioned waters regarding the values in the indices of nitrate, sulphate and hydrogenic ions, which are mainly related to the chemical composition of the ore mines, where the alkaline chemicals (Ca, K, Na, Mg, etc.) mainly predominate, and to the intensive agricultural activities carried out in the specific areas, particularly in the riverine land areas, as well as to the application of the single nitrogenous fertilization system and most likely to the development of animal husbandry.

The total intensity of the geochemical flow of the heavy metals (chemical elements) into the Sotk and Masrik rivers considerably fluctuates and varies depending on the exploitation of the Sotk mining site, changes in the volumes of the ore mine, their feeding, and the distance from the mining site.

Considering the fact that the waters of the mentioned rivers are used also for irrigation purposes and that their constituent heavy metals are endowed with the great ability to accumulate in the soils (deposited), and last but not least, their mobility is extremely reduced in the base (alkaline) medium, especially when the environmental reaction (pH) equals to 9, therefore, it is recommended that the managerial body of the mining industry should constantly increase the amount of lime (CaO) in the acidic drainage of the mining site provided that the water pH will hold up minimum within the range of 9. At the same time, taking into account the fact that certain heavy metals (selenium, wolfram, vanadium, molybdenum, etc.) are more active in the basic environment, it is necessary to install filters for their refinement.

The research results should be used to zone the surrounding areas of mining companies according to the deposition degree of heavy metals. Besides, to reduce the content of heavy metals in the agricultural crops grown in those areas the required technological approaches (phytomelioration, bioremediation, heat treatment of the harvested crop, canning, etc.) should be observed.

## Figures and Tables

**Table 1:** The agrochemical indices of the experimental plots and the tailings sludge.

N^o^	Sampling Depth, cm	%			pH	Content of Available Nutrients, mg in 100 g Soil		
		Hygro-Scopic Moisture Content	Humus	CaCO_3_		N	P_2_O_5_	K_2_O
1	0–20/tailings sludge/	6.90	-	12.10	7.45	-	-	-
2	20–45/experimental plots under the tailings/	5.12	1.27	10.79	7.35	0.85	0.67	13.02

**Table 2:** Contamination of the tailing’s layers and underlying soils with heavy metals, mg/kg in soil *.

N^o^	Sampling Depth, cm	Mo	Cu	Zn	Co	Ni	Pb
1	0–20 (tailings layer)	59.6	30.0	68.2	290	318.0	136.5
		3.8	18.0	16.0	18.0	46.0	32.0
2	20–45 (experimental plots layer)	6.20	15.0	24.20	5.00	15.4	1.040
		1.20	3.60	2.30	0.250	0.58	0.240

* Numerator shows total metal content, denominator shows the concentration of the mobile e form.

**Table 3:** The content of mobile forms of heavy metals in the soil of the experimental plot before crop planting per variants.

N^o^	Variants	Name of Heavy Metals					
		Pb	Ni	Cu	Zn	Co	As
1	Control (Without fertilization)	0.133	0.444	3.30	1.898	0.133	traces
2	Manure 40 t/ha-background	0.166	0.509	3.70	2.000	0.166	
3	Background + Zeolite 3 tons/ha	0.196	0.585	3.30	2.962	0.196	
4	Background + Zeolite 6 tons/ha	0.106	0.561	2.90	1.610	0.106	
5	Background + Zeolite 9 tons/ha	0.290	0.749	4.00	2.880	0.290	
6	Background + Bentonite 3 tons/ha	0.153	0.522	3.30	1.793	0.153	
7	Background + Bentonite 6 tons/ha	0.182	0.512	3.30	1.932	0.182	
8	Background + Bentonite 9 tons/ha	0.198	0.449	1.29	2.134	0.198	

**Table 4:** The effect of applying growing doses of natural mineral ameliorants on the background of manure on the crop yield capacity, c/ha.

N^o^	Variants	Potato						Bean (Green)						Eggplant		
		Per Replications			Average Yield	Yield Surplus		Per Replications			Average Yield	Yield Surplus		Average Yield	Yield Surplus	
		I	II	III		c/ha	%	I	II	III		c/ha	%		c/ha	%
1	Control (without fertilization)	98.0	102.0	101.8	100.6	-	-	63.0	71.0	67.0	67.0	-	-	87.0	-	-
2	Manure 40 t/ha-background	195.0	194.0	199.0	196.0	95.4	94.8	94.0	92.0	96.0	94.0	27.0	40.3	175.0	88.0	101.1
3	Background + Zeolite 3 t/ha	210.0	202.0	212.0	208.0	107.4	106.8	93.6	97.0	100.4	97.0	30.0	44.8	184.0	97.0	111.5
4	Background + Zeolite 6 t/ha	213.0	224.0	223.0	220.0	119.4	118.7	104.0	101.6	106.4	104.0	37.0	55.2	200.0	113.0	129.9
5	Background + Zeolite 9 t/ha	227.0	224.0	221.0	224.0	123.4	122.7	99.5	102.0	104.5	102.0	35.0	52.2	203.0	116.0	133.3
6	Background + Bentonite 3 t/ha	243.0	249.0	246.0	246.0	145.4	144.5	115.0	112.0	118.0	115.0	48.0	71.6	213.0	126.0	144.8
7	Background + Bentonite 6 t/ha	269.0	266.0	272.0	269.0	168.4	167.4	126.0	128.0	130.0	128.0	61.0	91.0	229.0	142.0	163.2
8	Background + Bentonite 9 t/ha	279.0	275	271.0	275.0	174.4	173.4	132.0	129.0	135.0	132.0	65.0	97.0	228.0	143.0	164.4
	Sx, %				4.2						2.4			3.1		
	LSD_0.95_, c				6.8						4.9			4.6		

**Table 5:** The effect of applying growing doses of natural mineral ameliorants on the background of manure on the crops qualitative indicators.

N^o^	Variants	Average Potato Yield	Starch Content, %	Nitrates Content, mg/kg	Starch Yield, c/ha	Average Eggplant Yield, c/ha	Nitrates Content, mg/kg	Green Bean Yield, c/ha	Nitrates Content, mg/kg
1	Control (without fertilization)	100.6	17.5	85.0	17.6	87.0	170.0	67.0	120
2	Manure 40 t/ha-background	196.0	16.0	175.0	31.4	175.0	235.0	94.0	190
3	Background + Zeolite 3 t/ha	208.0	16.2	165.0	33.7	184.0	240.0	97.0	180
4	Background + Zeolite 6 t/ha	220.0	15.9	170.0	35.0	200.0	225.0	104.0	185
5	Background + Zeolite 9 t/ha	224.0	15.7	168.0	35.2	203.0	230.0	102.0	175
6	Background + Bentonite 3 t/ha	246.0	16.2	185.0	39.9	213.0	245.0	115.0	200
7	Background + Bentonite 6 t/ha	269.0	17.0	180.0	45.7	229.0	240.0	128.0	205
8	Background + Bentonite 9 t/ha	275.0	16.6	185.0	45.7	238.0	235.0	132.0	215

**Table 6:** The effect of applying growing doses of natural mineral ameliorants on the background of manure on the accretion/accumulation of heavy metals in the yield of potato tubers and green beans (mg/kg in dry matter).

N^o^	Variants	Potato						Bean					
		Pb	Ni	Cu	Zn	Co	As	Pb	Ni	Cu	Zn	Co	As
1	Control (Without fertilization)	<0.01	0.320	2.50	1.862	0.066	traces	<0.01	0.29	1.20	1.775	0.031	traces
2	Manure 40 t/ha-background	<0.01	0.317	2.43	1.859	0.076		<0.01	0.30	1.16	1.876	0.029	
3	Background + Zeolite 3 t/ha	<0.01	0.270	2.00	1.657	0.035		<0.01	0.51	0.95	1.880	0.018	
4	Background + Zeolite 6 t/ha	<0.01	0.200	2.00	1.670	0.039		<0.01	0.52	0.93	1.800	0.020	
5	Background + Zeolite 9 t/ha	<0.01	0.200	1.80	1.540	0.035		<0.01	0.41	0.95	2.750	<0.01	
6	Background + Bentonite 3 t/ha	<0.01	0.250	1.20	1.500	0.031		<0.01	0.36	0.68	1.650	<0.01	
7	Background + Bentonite 6 t/ha	<0.01	0.250	0.95	1.450	0.030		<0.01	0.38	0.67	1.600	<0.01	
8	Background + Bentonite 9 t/ha	<0.01	0.270	0.95	1.450	0.025		<0.01	0.48	0.65	1.620	<0.01	

**Table 7:** Organoleptic and chemical indicators of the Sotk river waters depend on the exploitation of the Sotk gold mine.

N^o^	Indicators	Measuring Unit	October–November, 2010				October–November, 2015				November, 2019			
			The Indices of Cellar Pit, October–November	1.0 km above the Sotk Community	Opposite the Sotk Community	River Mouth (Frith)	The Indices of Cellar Pit, October-November	1.0 km above the Sotk Community	Opposite the Sotk Community	River Mouth (Frith)	The Indices of Cellar Pit, October–November	1.0 km above the Sotk Community	Opposite the Sotk Community	River Mouth (Frith)
1	Hydrogenic index		8.30	8.20	8.40	8.74	8.40	8.36	8.44	8.40	8.43	8.37	8.35	8.78
2	Dissolved oxygen	mg/L	10.20	9.20	6.40	10.30	9.8	6.40	7.60	6.60	7.20	6.80	9.30	5.40
3	BOD_5_	mgO_2_/L	1.80	2.00	1.30	2.20	2.0	2.15	2.18	2.10	1.79	1.00	2.70	1.91
4	BOD	mgO/L	-	-	18.0	-	15.40	28.0	15.0	27.0	15.0	35.00	17.00	30.0
5	Total phosphorus	mg/L	0.02	0.01	0.05	0.00	0.03	0.05	0.01	0.05	0.02	0.02	0.01	0.04
6	Ammonium ion	mgN/L	0.30	0.25	0.19	0.18	0.41	0.14	0.09	0.14	0.23	0.12	0.14	0.19
7	Nitrite ion	mgN/L	0.01	0.00	0.01	0.01	0.06	0.04	0.03	0.03	0.00	0.00	0.01	0.04
8	Nitrate ion	mgN/L	0.00	-	1.31	-	0.01	2.23	0.26	2.18	0.37	0.45	3.46	2.44
9	Phosphate ion	mg/L	0.05	0.05	0.10	0.05	0.07	0.18	0.03	0.17	0.00	0.01	0.17	0.07
10	Chloride ion	mg/L	2.65	2.34	2.31	8.30	2.45	5.26	1.62	5.13	2.43	3.27	7.96	7.58
11	Sulphate ion	mg/L	20.40	21.64	28.45	65.11	19.62	30.30	8.46	29.30	6.80	15.75	41.53	43.37
12	Total dissolved salts	mg/L	239.0	246.4	306.0	299.6	261.4	330.0	202.0	309.0	210.0	230.8	285.0	253.5
13	Electroconductivity	mSm/cm	829.0	379.0	470.0	461.0	972.4	507.0	311.0	502.0	323.0	355.0	439.0	390.0
14	Suspended particles	mg/L	6.90	2.70	15.40	1.60	7.80	64.40	5.90	62.40	3.50	11.50	47.50	14.1
15	Permeability	cm	29	29	30	30	27	29	29	29	26	27	28	28
16	Color (visual)	degree	4	4	4	3	4	4	5	4	4	4	4	4
17	Flavor	point	Light flavor 2	2	2	2	3	2	2	3	3	2	2	3

**Table 8:** Organoleptic and chemical indicators of the Masrik river waters depending on the exploitation of the Sotk gold mine.

N^o^	Indicators	Measuring Unit	October–November, 2010			October–November, 2015			November, 2019				
			1.5 km above the Community of V. Shorzha	1.5 km below the Community of V. Shorzha	River Mouth	1.5 km above the Community of V. Shorzha	1.5 km below the Community of V. Shorzha	River Mouth	The Mouth (frith) of the Karchaghbyur River	1.5 km above the Community of V. Shorzha	1.5 km below the Community of V. Shorzha	River Mouth	0.5 km below the River Mouth after Interflowing
1	Hydrogenic index		9.20	9.00	8.49	8.50	8.41	8.14	7.3	7.88	8.38	8.24	7.55
2	Dissolved oxygen	mg/L	11.40	11.2	10.00	8.90	7.00	7.30	7.9	7.70	4.10	6.70	6.90
3	BOD_5_	mgO_2_/L	2.80	2.4	2.80	1.62	1.00	1.33	1.2	1.50	1.05	2.60	2.15
4	BOD	mgO/L	-	8	5.00	8.00	11.4	18.0	10.4	10.0	30.00	15.00	20.0
5	Total phosphorus	mg/L	0.01	0.0061	0.1274	0.07	0.09	0.16	0.01	0.03	0.03	0.12	0.27
6	Ammonium ion	mgN/L	0.10	0.1012	0.1944	0.06	0.06	0.07	0.21	0.06	0.11	0.08	0.14
7	Nitrite ion	mgN/L	0.08	0.092	0.0276	0.01	0.03	0.06	0.13	0.00	0.00	0.03	0.11
8	Nitrate ion	mgN/L	-	0.5534	0.4944	0.33	0.56	0.93	0.03	0.26	0.76	1.43	2.76
9	Phosphate ion	mg/L	0.01	0.0142	0.3390	0.40	0.39	0.37	0.12	0.04	0.07	0.28	0.23
10	Chloride ion	mg/L	4.35	4.4061	4.6562	3.06	2.62	2.41	1.10	1.19	2.15	3.09	4.92
11	Sulphate ion	mg/L	11.61	11.69	16.54	5.16	5.81	6.96	10.4	4.06	6.96	15.01	20.97
12	Total dissolved salts	mg/L	152.1	152.60	156.6	154.0	162.6	176.00	114.2	69.0	146.90	141.00	171.00
13	Electroconductivity	mSm/cm	234.0	230.60	241.0	236.0	247.4	271.00	118.2	106.0	226.00	218.00	267.00
14	Suspended particles	mg/L	1.80	1.87	15.80	60.20	23.60	18.50	2.14	5.50	19.90	28.70	19.90
15	Permeability	cm	31	31	30	30	32	31	31	32	31	32	32
16	Color (visual)	degree	3	3	4	4	4	4	4	3	3	3	3
17	Flavor	point	Light flavor 2	2	3	3	With flavor 4	4	3	3	3	3	3

**Table 9:** Content of individual chemical elements in the waters of the Sotk river by dates.

N^o^	Name of the Chemical Elements	Measuring Unit	October–November, 2010			October–November, 2015			November, 2019			
			Above the Sotk Community	Opposite the Sotk Community	River Mouth	Above the Sotk Community	Opposite the Sotk Community	River Mouth	Above the Sotk Community	Opposite the Sotk Community	River Mouth	After Interflowing of the Sotk and Masrik Rivers
1	Ca	mg/L	35.09	40.07	50.97	37.91	21.87	28.15	20.35	23.02	46.04	34.32
2	Mg	mg/L	33.63	39.91	29.26	42.14	31.12	30.72	32.56	41.09	19.70	30.39
3	K	mg/L	1.18	1.68	5.49	2.77	0.52	5.47	0.48	0.93	2.07	2.45
4	Na	mg/L	3.06	6.07	11.38	3.83	2.68	10.86	2.72	6.35	7.58	11.70
5	Al	mg/L	0.06	0.03	0.04	0.06	0.02	0.56	0.02	0.02	0.12	0.14
6	Se	mg/L	0.00	0.00	0.01	0.00	0.00	0.00	0.00	0.00	<0.00	0.00
7	S/Sulphur	mg/L	0.00	0.00	0.00	0.00	0.00	0.01	0.00	0.00	0.00	0.01

**Table 10:** Content of individual chemical elements in the waters of the Masrik river by dates.

N^o^	Name of the Chemical Elements	Measuring Unit	October–November, 2010			October–November, 2015			November, 2019			
			1.5 km above the Community of V. Shorzha	1.5 km below the Community of V. Shorzha	River Mouth	1.5 km above the Community of V. Shorzha	1.5 km below the Community of V. Shorzha	River Mouth	1.5 km above the Community of V. Shorzha	1.5 km below the Community of V. Shorzha	River Mouth	After Interflowing of the Sotk and Masrik Rivers
1	Ca	mg/L	32.43	28.4	26.42	31.96	30.89	30.89	16.45	30.03	28.75	29.36
2	Mg	mg/L	6.61	8.40	9.16	5.82	8.86	8.86	2.31	6.78	7.55	11.22
3	K	mg/L	2.29	3.00	3.30	4.42	4.46	4.46	0.64	2.60	2.95	4.48
4	Na	mg/L	9.04	9.11	9.11	7.86	9.89	9.89	3.33	10.46	5.36	11.92
5	Al	mg/L	0.01	0.03	0.04	0.79	0.06	0.06	0.13	0.11	0.15	0.23
6	Se	mg/L	0.00	0.00	0.00	0.00	0.00	0.00	0.00	0.00	0.00	0.00
7	S (Sulphur)	mg/L	0.00	0.00	0.00	0.00	0.00	0.00	0.00	0.00	0.00	0.00

**Table 11:** Average indices of heavy metals in Sotk, Masrik, and Karchaghbyur rivers and arable lands irrigated by those waters.

Name of the Element	Name of the River and Basin						Phytotoxic Concentrations of Heavy Metals in Soil, mg/kg [10]
	Sotk		Masrik		Karchaghbyur		
	in Waters, g/L	in Arable Land mg/kg	in Waters, g/L	in Arable Land mg/kg	in Waters, g/L	in Arable Land mg/kg	
Ag	<10^−5^	0.2	<10^−5^	0.8	-	-	2
As	0.080	14.3	0.045	17.0	-	0.012	15–50
Cd	<10^−3^	0.7	<10^−2^	0.9	-	-	3–8
Co	<10^−4^	10.0	<10^−5^	13.4	-	-	25–50
Cr	0.0080	4.6	0.0065	20.0	-	-	75–100
Cu	0.035	10.5	0.027	19.2	0.001	1.0	60–125
Hg	0.003	0.2	0.0015	0.3	-	-	0.3–5
Mn	61.3	980.0	59.4	640.0	-	traces	1500–3000
Ni	0.003	48.0	0.009	45.0	-	-	100
Pb	0.007	21.0	0.009	27.0	0.00004	0.7	100–120
Zn	0.020	90.0	0.87	78.0	0.0003	1.2	70–200
Sb	<10^−4^	2.0	<10^−3^	6.4	-	-	5–10
Se	<10^−5^	-	<10^−4^	0.7	-	-	5–10
V	0.035	1.4	0.026	3.0	-	-	5–100

## Data Availability

The datasets generated and analyzed during the current study are available from the corresponding author upon reasonable request.
